# An integrated prognosis model of pharmacogenomic gene signature and clinical information for diffuse large B-cell lymphoma patients following CHOP-like chemotherapy

**DOI:** 10.1186/s12967-020-02311-1

**Published:** 2020-03-30

**Authors:** Jinglei Hu, Jing Xu, Muqiao Yu, Yongchao Gao, Rong Liu, Honghao Zhou, Wei Zhang

**Affiliations:** 1 Department of Clinical Pharmacology, Xiangya Hospital, Central South University, 87 Xiangya Road, Changsha, 410008 People’s Republic of China; 2 Institute of Clinical Pharmacology, Central South University, Hunan Key Laboratory of Pharmacogenetics, 110 Xiangya Road, Changsha, 410078 People’s Republic of China; 3 Engineering Research Center of Applied Technology of Pharmacogenomics, Ministry of Education, 110 Xiangya Road, Changsha, 410078 People’s Republic of China; 4 National Clinical Research Center for Geriatric Disorders, 87 Xiangya Road, Changsha, 410008 Hunan People’s Republic of China; 5 Xiangya School of Stomatology, Central South University, Changsha, 410078 Human People’s Republic of China

**Keywords:** Diffuse large B-cell lymphoma, Pharmacogenomic signature, Survival, CHOP-like chemotherapy

## Abstract

**Background:**

As the most common form of lymphoma, diffuse large B-cell lymphoma (DLBCL) is a clinical highly heterogeneous disease with variability in therapeutic outcomes and biological features. It is a challenge to identify of clinically meaningful tools for outcome prediction. In this study, we developed a prognosis model fused clinical characteristics with drug resistance pharmacogenomic signature to identify DLBCL prognostic subgroups for CHOP-based treatment.

**Methods:**

The expression microarray data and clinical characteristics of 791 DLBCL patients from two Gene Expression Omnibus (GEO) databases were used to establish and validate this model. By using univariate Cox regression, eight clinical or genetic signatures were analyzed. The elastic net-regulated Cox regression analysis was used to select the best prognosis related factors into the predictive model. To estimate the prognostic capability of the model, Kaplan–Meier curve and the area under receiver operating characteristic (ROC) curve (AUC) were performed.

**Results:**

A predictive model comprising 4 clinical factors and 2 pharmacogenomic gene signatures was established after 1000 times cross validation in the training dataset. The AUC of the comprehensive risk model was 0.78, whereas AUC value was lower for the clinical only model (0.68) or the gene only model (0.67). Compared with low-risk patients, the overall survival (OS) of DLBCL patients with high-risk scores was significantly decreased (HR = 4.55, 95% CI 3.14–6.59, log-rank p value = 1.06 × 10^−15^). The signature also enables to predict prognosis within different molecular subtypes of DLBCL. The reliability of the integrated model was confirmed by independent validation dataset (HR = 3.47, 95% CI 2.42–4.97, log rank p value = 1.53 × 10^−11^).

**Conclusions:**

This integrated model has a better predictive capability to ascertain the prognosis of DLBCL patients prior to CHOP-like treatment, which may improve the clinical management of DLBCL patients and provide theoretical basis for individualized treatment.

## Background

Diffuse large B-cell lymphoma (DLBCL), as the most frequent adult non-Hodgkin lymphoma, representing approximately 30–40% of patients with lymphoid neoplasms in different geographic regions, with an annual incidence rate of over 100,000 cases worldwide [1, 2]. As an aggressive malignancy, DLBCL showing some heterogeneous disorders with variable clinical characteristics, morphology, gene profile, and response to therapy [3, 4]. These changeable characters make it difficult to prognosis and decide therapeutic strategies for patients with DLBCL. Nowadays, the initial standard therapy with rituximab in combination with CHOP (R-CHOP) chemotherapy is consists of rituximab, cyclophosphamide, doxorubicin, vincristine and prednisone, and approximately 75–80% patients get completely remission [5, 6]. However, a proportion of patients still intractable to this first-line therapy. Therefore, it is increasingly essential to identify early therapeutic prognosis and identify high-risk patients who are unlikely to respond sensitively or benefit from therapeutic medicines.

The resistance of chemotherapy is changing among patients. It often occurs at the beginning of drug treatment or after an initial response. Numerous clinical, pathological and molecular markers have been developed to characterize the disease. Different kinds of resistance have been defined and associated with adverse clinical prognosis. Patients response to cancer medicines is significantly related with the genetic features of cancer cells lines [7]. Due to the heterogeneous response of patients to therapies and the frequent development of drug resistance, it is vital to recognize the molecular biomarkers to manage tolerable treatments for patients [8–10]. Since the development of microarrays, resistance gene signatures (REGSs) have been widely researched for prediction of cancer chemoresistance. There are some useful websites which contain plenty of information about drug resistance in cancer cell lines [11–13]. Genomics of Drug Sensitivity in Cancer (GDSC), one of the public databases provides drug sensitivity information in cancer cell lines and link with genomic datasets to identify new biomarkers for therapy. Recently, many molecular studies involving gene expression profiling in cancer cell lines [14–16] have shown that variant resistance possibility of therapies could influence patients’ prognosis. Researches on drug resistance based on cancer cell lines have typically been generated through dose response experiments. Based on various analysis methods, more useful REGSs have been developed with strong prediction for clinical outcomes. Boegsted et al. [15], Steffen et al. [14] and Liedtke et al. [16] have described predictive REGSs based on the gene expression of cells lines for multiple myeloma, DLBCL and breast cancer, respectively.

On the purpose of predicting response to cyclophosphamide, doxorubicin and vincristine, Steffen et al. [14] have formulated three drug-specific resistance gene signatures from DLBCL patients treated with CHOP-like therapy, which could be potentially used to guide effective therapy for the individual patient from the beginning. These discovery and application of prognostic molecular signatures help identify potentially high-risk patients with DLBCL. Currently, in addition to this molecular methods, bio-clinical approach is also used to formulate treatment modalities for DLBCL [17, 18]. There are many clinicopathological features associated with the outcomes of DLBCL patients [19–21], such as the Eastern Cooperative Oncology Group (ECOG) performance status, age, Ann Arbor stage and the nodal and extra nodal sites number were used commonly as clinical parameters in large number of studies. International Prognostic Index (IPI) is a robust prognostic tool, based on clinical and biochemical pre-treatment parameters, usually used to predict the outcomes of patients with DLBCL [22]. However, it seems that REGSs or clinical factors alone are insufficient to predict patients’ prognosis. And to data, there is no one has ever considered to combine drug resistance signatures with clinical information to predicting prognosis of DLBCL. Thus, in order to establish a more sensitive models for forecasting patients’ outcomes prior to therapy, our model comprehensively considered both drug sensitive information and clinical factors which have closely relation to the prognosis.

In our study, to build an integrated model for prognosis prediction for DLBCLs, we initially calculated cyclophosphamide, doxorubicin and vincristine drug resistance probabilities for each DLBCL patients treated with CHOP-like (including cyclophosphamide, doxorubicin, vincristine) chemotherapy. Then we analyzed clinical data and three drug resistance probabilities of DLBCL patients treated with CHOP-like therapy from GEO to screen factors that predictive for survival time, such as OS or progression-free survival (PFS). By using Cox regression analysis, we found four clinical variables and the resistance probabilities to doxorubicin and vincristine were associated with OS of DLBCL patients, and developed an integrated gene-and-clinical model for OS and another integrated gene-and-clinical model for PFS including three clinical variables and the resistance probabilities to doxorubicin from the training dataset, respectively. Due to the PFS of patients in the validating dataset wasn’t recorded, we only confirmed the prognostic prediction ability of model for OS in validation dataset. We also compared the prognosis power of this integration model with a pharmacogenomic signature-alone model and a clinical-alone model. Our results suggested that a pharmacogenomic gene-and-clinical signature can more exactly indicates the prognosis of patients with DLBCL before chemotherapy.

## Methods

### Patients’ samples

Two datasets that containing corresponding clinical information and genome-wide gene expression microarray data by using pretreatment lymphoma tissues from DLBLC were download from the GEO databases (http://www.ncbi.nlm.nih.gov/geo/, Additional file 1: Table S1). All patients receiving CHOP or CHOP based (R-CHOP) treatment (cyclophosphamide, doxorubicin, vincristine and prednisone plus rituximab). The gene expression profiles of patients were conducted using the Affymetrix Human Genome U133 Plus 2.0 array from datasets utilized in this study. Our study included overall 791 patients with DLBCL, consisting of 449 patients from Visco’s study (GSE31312) [23] and 342 patients from Lenz’s study (GSE10846) [24], who have complete clinical information. Dataset GSE31312 was used for train prognosis models, and GSE10846 was used to validate the prognosis model generated from the training dataset. The clinical endpoint was death, or the date of last assessment without any such event (censored observation), and the secondary endpoint was disease progression. The raw files in CEL format were downloaded from GEO and preprocessed with the RMA algorithm using the ‘affy’ R package for the expression data. The probe-level expression values were converted into gene-based expressions via the collapse row function [25].

### Clinical and Pharmacogenomic factors

Univariate cox regression analysis was separately performed to select the demographic and clinical features and drug resistance signatures associated with the OS or PFS of DLBCL patients from following eight factors: gender, age at diagnosis, stage, extra nodal sites number, ECOG performance score and resistance probabilities of cyclophosphamide, doxorubicin, and vincristine. REGSs for three specific drugs (cyclophosphamide, doxorubicin, and vincristine) were applied to calculated the possibility of drug-specific resistance for individual patient [14]. Next, by using elastic net-regulated Cox regression, clinical related factors or pharmacogenomic factors resulting in a p-value less than 0.05 were respectively selected into the clinical alone model or gene alone model in the training dataset with 1000 times cross validations using the glmnet R package.

### Integrated clinical-and-gene model for OS

Furthermore, we built an integrated model for OS of DLBCL patients with six factors, comprising age at diagnosis, stage, extra nodal sites number, the ECOG performance score, drug resistance probability of doxorubicin and drug resistance probability of vincristine. The prediction score was calculated according the following formula:

$$Prognosis\;score\; \left( {OS} \right) = \mathop \sum \limits_{i = 1}^{n} \left( {factor_{i} *Coe_{i} } \right)$$

where n is six, factor_i_ and Coe_i_ stands for the value of factor_i_ and the estimated regression coefficient of factor_i_ in the elastic net-regulated Cox regression model after 1000 times cross validation, respectively. We used multivariate Cox regression analysis to examine whether drug specific resistance signatures were independent of clinical factors.

### Integrated clinical-and-gene model for PFS

Meanwhile, we built an integrated model for PFS of DLBCL patients with 4 factors, comprising stage, extra nodal sites number, the ECOG performance score, drug resistance probability of doxorubicin. The prediction score was calculated according the following formula:

$$Prognosis\;score\; \left( {PFS} \right) = \mathop \sum \limits_{i = 1}^{n} \left( {factor_{i} *Coe_{i} } \right)$$

where n is four, factor_i_ and Coe_i_ stands for the value of factor_i_ and the estimated regression coefficient of factor_i_ in the elastic net-regulated Cox regression model after 1000 times cross validation, respectively. We used multivariate Cox regression analysis to examine whether drug specific resistance signatures were independent of clinical factors.

### Signature performance evaluation

According to the median cutoff of the signature, we classified DLBLC patients into two distinct risk groups. Then DLBCL patients’ OS or PFS were compared between two groups via Kaplan–Meier curves and p values (log-rank test). ROC analysis was used to estimate the prognostic utility of this integrated signatures according to their ability and efficiency to predict the risk of death or disease progress, which was performed with survivalROC R packages. The ROC curve draws the true-positive vs. false-positive predictions. The higher AUC value indicates better prediction efficiency (AUC = 0.5 means random prediction). The predictive capability of the integrated model was compared with the pharmacogenomic gene model and the clinical model. Meanwhile, to estimate the prediction and survival classification value of integrated model, the overall concordance statistic (C-index), integrated discrimination improvement (IDI), net reclassification improvement (NRI) and restricted mean survival (RMS) ratio were calculated in training and validating dataset. Furthermore, we examined the predictive power of prognostic model in distinct DLBCL subtypes.

### Statistical analysis

Statistical analysis in this study were all performed using the R software version 3.4.1 (https://www.r-project.org/) with related packages or our custom compiled functions. All reported p-values were two sided. The survival R package was utilized for survival analysis. We calculated the hazard ratio (HR) and 95% confidence interval (CI) with a Cox regression model, and survival curves were drawn from Kaplan–Meier estimates. Differences in survival between groups were compared using the two-sided log-rank test. C-index, IDI and NRI were calculated in each datasets by R package survC1. The RMS analysis was performed using R package survRM2 [26].

## Results

### Demographic and clinical characteristics of datasets utilized in our study

DLBCL datasets with whole genome mRNA expression information and clinical data were obtained from the GEO database with accession number GSE31312 and GSE10846. After removal of patients without clinical and survival data, 791 DLBCL patients were analyzed in our study (Table 1), including 449 patients from GSE31312 and 342 patients from GSE10846. Gene expression of DLBCL patients were profiled with Affymetrix HG U133 plus 2.0 gene chips. Patient characteristics, including age at diagnosis, gender, extra nodal sites number, tumor stage, ECOG performance score, molecular subtypes and overall survival, are listed in Table 1. Five clinical factors were analyzed by univariate survival analysis, including gender, age at diagnosis, stage, the number of extra nodal sites, the ECOG performance score. We determined all of the above tested clinical variable except gender to be correlated with OS in DLBCL patients (p < 0.05, Additional file 2: Table S2). And stage, the number of extra nodal sites, the ECOG performance score were related to PFS in DLBCL patients (p < 0.05, Additional file 3: Table S3).

**Table 1:** Clinical and pathological characteristics of patients with DLBCL in our study

Characteristic	Training dataset	Validating dataset
	(GSE31312)	(GSE10846)
Sample size	449	342
Age, years mean (SD)	61.81 (14.75)	61.24 (15.48)
Gender		
Female	189	144
Male	260	182
Unknown	0	16
Stage		
1	124	58
2	96	103
3	101	77
4	128	104
Unknown	0	0
≥ 2 Extranodal sites	100	29
ECOG^a^ performance status > 1	81	84
Microarray subtype		
GCB	214	147
ABC	193	144
Unclassified subtype	42	51
Unknown	0	0
Overall survival		
Time, years mean (SD)	3.24 (2.14)	2.80 (2.35)
Death	164	148

*GCB* germinal center B-cell-like subtype, *ABC* activated B-cell-like subtype, *SD* standard deviation

^a^The Eastern Cooperative Oncology Group (ECOG) performance score ranges from 0 to 3, with a higher score indicating greater impairment

### Pharmacogenomic gene only signature

In dataset GSE31312 and GSE10846, each individual was calculated three drug-specific resistance probability for cyclophosphamide, doxorubicin, and vincristine separately via resistance gene signature classifiers [14]. Among 3 drug-specific resistance probability, two of them with elastic net-regulated Cox regression coefficients that did not equal 0 were included in the pharmacogenomic gene signature for OS and one drug-specific resistance probability was chosen as pharmacogenomic gene signature for PFS. The risk-score formula based on the expression of resistance gene signature for death risk prediction as follows: pharmacogenomic gene score = coefficient* (expression level of gene level), the corresponding coefficient for each gene is listed in Additional file 4: Table S4 (OS) and Additional file 3: Table S3 (PFS). According to the formula of model for OS or PFS, patients in the training dataset were divided into two adverse risk groups via the median score as the cut-off value, respectively. The survival time between two groups were estimated by using Kaplan–Meier analysis. Then using the two-sided log rank test to compare different OS or PFS between these two risk groups in the training and validating dataset (Additional file 5: Figure S1; Additional file 6: Figure S2). In univariate factor analysis, the HR of the high-risk group versus the low-risk group for OS was 1.79 (95% CI 1.30-2.46, *p *= 3.50 × 10^−4^) in the training dataset and 1.64 (95% CI 1.17-2.30, *p *= 3.77 × 10^−3^) in the validation dataset and for PFS was 1.71 (95% CI 1.26–2.32; *p *= 6.26 × 10^−4^) in the training dataset, suggesting that the higher risk scores based on pharmacogenomic gene signature was markedly related to shorter OS or PFS.

### Prognostic value of the drug resistance factors is independent of clinical information

To further test whether the drug resistance signature was an independent prognosis predictor of DLBCL patients, the multivariate analysis was performed. We first performed Multivariate Cox regression analysis and identified clinical information that be associated with OS (Table 2) or PFS (Additional file 7: Table S5). Further, the effect of above predictors on OS or PFS of DLBCL patients was further estimated by multivariate Cox regression analysis. The results showed that resistance probability of doxorubicin is an independent predictor of OS when adjusted by age, extra nodal sites number, stage and ECOG score either in training dataset (HR = 2.70, 95% CI 1.24–5.86, *p *= 1.23 × 10^−2^) and validating dataset (HR = 3.24, 95% CI 1.41–7.45, *p *= 5.61 × 10^−3^), and is also an independent predictor of PFS when adjusted by extra nodal sites number, stage and ECOG score in training dataset (HR = 3.29, 95% CI 1.54–7.05, *p* = 2.14 × 10^−3^).

**Table 2:** Multivariate Cox regression analysis of overall survival in each dataset

	Multivariate analysis		
	HR	95% CI	p value
Training cohort			
GSE31312 (n = 449)			
Age	1.03	1.01–1.04	1.38 × 10^−5^
Extra nodal sites number	1.20	1.03–1.41	2.37 × 10^−2^
Stage	1.31	1.12–1.52	6.26 × 10^−5^
ECOG^a^	1.41	1.21–1.64	7.33 × 10^−6^
Resistance probability of doxorubicin	2.70	1.24–5.86	1.23 × 10^−2^
Resistance probability of vincristine	1.28	0.61–2.70	0.51
Validating cohort			
GSE10846 (n = 342)			
Age	1.02	1.01–1.04	1.45 × 10^−4^
Extra nodal sites number	0.99	0.80–1.22	0.95
Stage	1.41	1.19–1.67	5.40 × 10^−5^
ECOG^a^	1.59	1.32–1.91	6.43 × 10^−7^
Resistance probability of doxorubicin	3.24	1.41–7.45	5.61 × 10^−3^
Resistance probability of vincristine	1.40	0.61–3.23	0.43

*HR* hazard ratio, *95% CI* 95% confidence interval

^a^The Eastern Cooperative Oncology Group (ECOG) performance score ranges from 0 to 3, with a higher score indicating greater impairment

### Integrated clinical-and-gene model in the training dataset

**Fig. 1:**
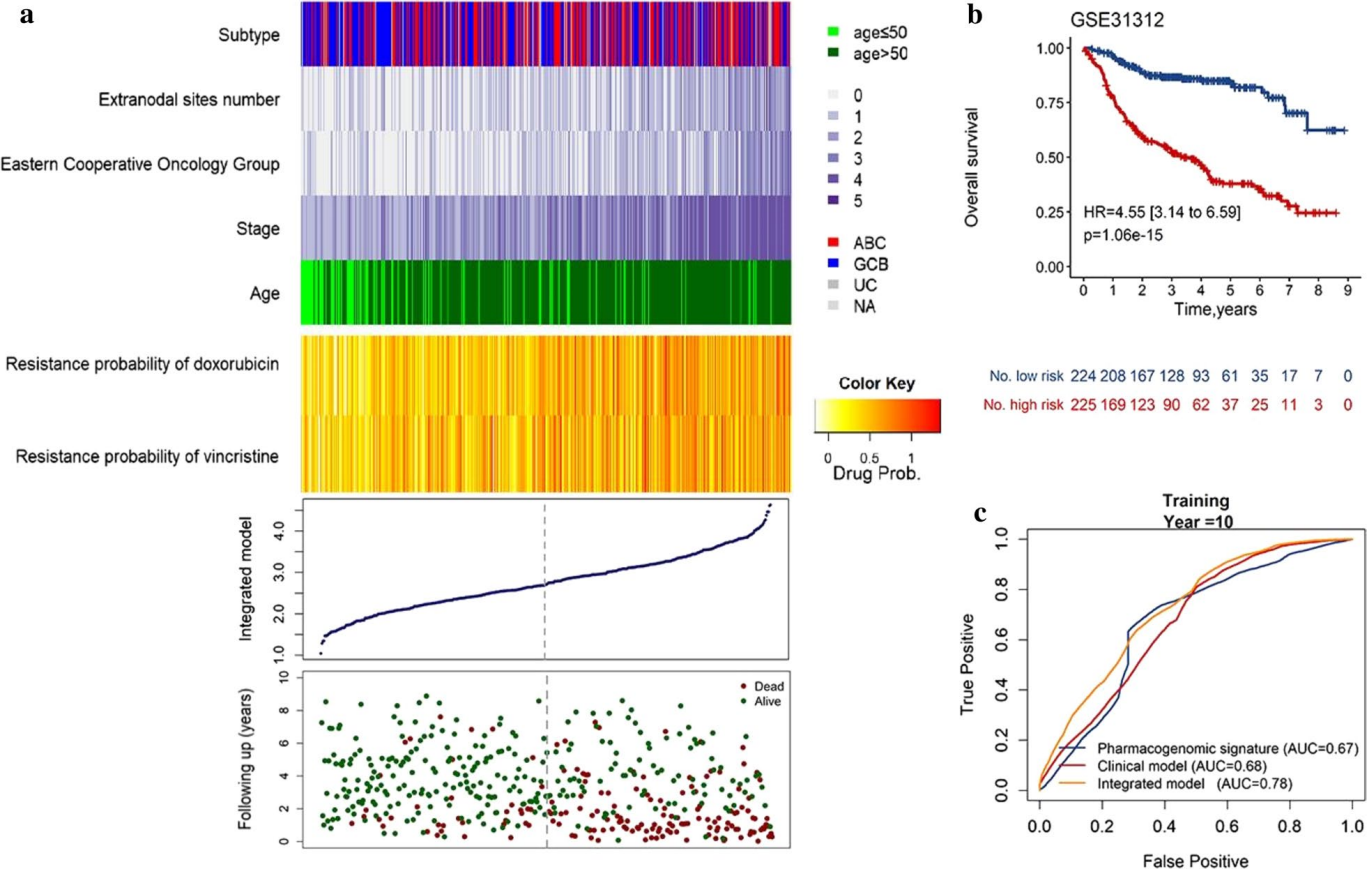
Integrated model analysis for OS of patients in the training dataset. Patients’ survival and disease progress status and risk score generated with integrated model were analyzed in the training set patients (GSE31312, n = 449). **a** The distribution plot, patients’ overall survival status and time and heatmap of the integrated model profiles. Rows represent clinical information and drug resistance probability, and columns represent patients. The grey dotted line represents the median integrated model risk score cutoff dividing patients into low- and high-score groups. Kaplan–Meier analysis for OS (**b**) of DLBLC patients using the integrated model in the training dataset. The ROC curves of the pharmacogenomic gene signature, clinical only model and integrated model for prediction of OS (**c**)

In our study, we develop an integrated clinical-and-genomic model for OS or PFS in the training dataset using elastic net-regulated Cox regression model after 1000 times cross validation, respectively. All of the prognosis related factors with a non-zero elastic net-regulated Cox regression coefficients were selected into the model for OS: drug resistance signature, age at diagnosis, stage, the number of extra nodal sites and the ECOG performance score (Table 3; Additional file 8: Figure S3). We created a risk-score formula for OS as follows: Prognosis score (OS) = 0.0213* (age at diagnosis) +0.1499* (extra nodal sites number) + 0.2360* stage + 0.2951* (ECOG performance score) + 0.8221* (drug resistance probability of doxorubicin) + 0.0998* (drug resistance probability of vincristine). According to prognosis score, patients were separated into low-risk (n = 224) and high-risk groups (n = 225) in the training dataset via the median score from formula as cut-off. Distribution of the prognosis score, survival status and gene expression in patients in the training dataset (Fig. 1a). In univariate factors analysis, the HR of high score group versus the low score group for OS was 4.55 (95% CI 3.14–6.59, *p* = 1.06 × 10^−15^, by log-rank test, Fig. 1b), suggesting that the patients with high scores was significantly associated with shorter OS. Meanwhile, A risk-score formula for PFS was developed as follows: Prognosis score (PFS) = 0.0726* (extra nodal sites number) + 0.3347* stage + 0.1615* (ECOG performance score) + 0.9754* (drug resistance probability of doxorubicin) (Additional file 9: Table S6; Additional file 10: Figure S4).

**Table 3:** Univariate cox regression analysis of overall survival and ridge regression coefficients of clinical information and drug resistance signatures in the training dataset

Clinical information	Univariate analysis			Coefficient
	HR	95% CI	p value	
Age	1.03	1.01–1.04	9.56 × 10^−6^	0.0213
Gender (reference = female)				
Male	0.95	0.70–1.30	0.75	–
Extra nodal sites number	1.43	1.25–1.63	1.38 × 10^−7^	0.1499
Stage	1.52	1.32–1.75	3.15 × 10^−9^	0.2360
ECOG^a^	1.54	1.34–1.77	1.66 × 10^−9^	0.2951
Drug resistance probability				
Cyclophosphamide	0.76	0.40–1.44	0.40	–
Doxorubicin	3.44	1.86–6.36	7.86 × 10^−5^	0.8221
Vincristine	2.41	1.31–4.41	4.48 × 10^−3^	0.0998

^a^The Eastern Cooperative Oncology Group (ECOG) performance score ranges from 0 to 3, with a higher score indicating greater impairment

*HR* hazard ratio, *95% CI* 95% confidence interval

To compare the sensitivity and utility of prognosis prediction between integrated gene-clinical model and pharmacogenomic gene only or clinical only model in the training model, Kaplan–Meier curve analysis and ROC analysis was conducted and the under receiver operating characteristic (AUC) value were calculated. ROC curves suggested that all the above three signatures showed prediction power in predicting OS in the training dataset (AUC > 0.5). Furthermore, compared with pharmacogenomic gene-only and clinical-only model, the integrated model with pharmacogenomic gene score and clinical information showed better performance in predicting OS at 10-year time point, as demonstrated by AUC values (Fig. 1c, Additional file 11: Table S7; Additional file 5: Figure S1). Compared with clinical-only model and pharmacogenomic gene signature, integrated model for OS had a highest mean C-index 0.73 with a SE of 0.02 and a significant RMS time ratio (1.73) in training dataset (Additional file 12: Table S8; Additional file 13: Table S9).

IPI, as a powerful predictor, is often used to predict outcomes in patients with DLBCL. According to risk group defined by our risk score and IPI, then we performed the Kaplan–Meier curve analysis to compare the value of survival prediction between these two predictive models in training dataset (Additional file 14: Figure S5).The integrated model achieved higher HR value (HR = 5.07) than IPI index (HR = 2.94), indicating that the predictive ability of our integrated model was better than IPI index.

### Validation of the prognostic integrated model for OS in independent dataset

**Fig. 2:**
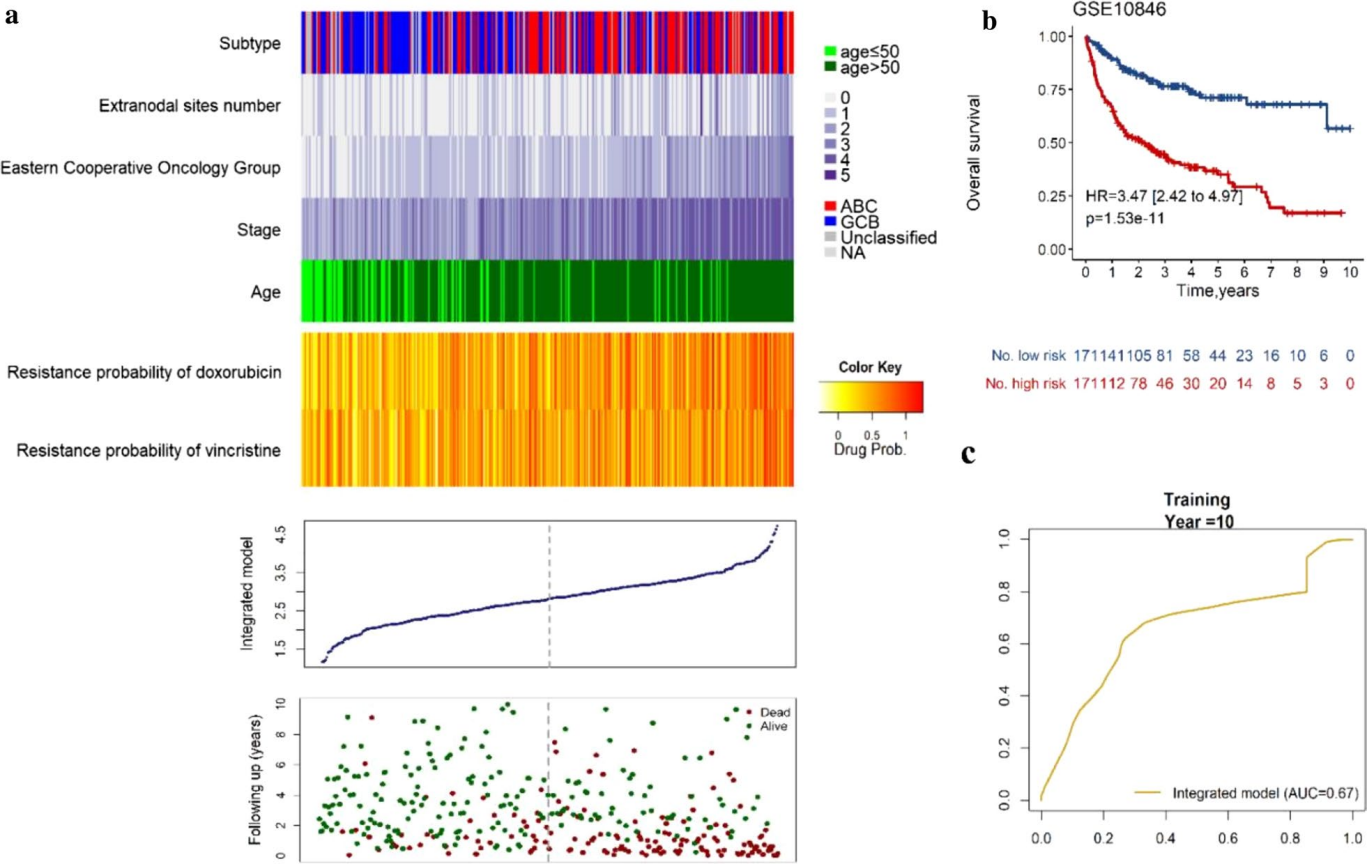
Performance evaluation of the integrated model for OS of DLBCL patients treated with CHOP-based chemotherapy in the validating dataset. Patients’ overall survival status and risk score generated with integrated model in the validating dataset (GSE10846, n = 342). **a** The distribution plot, patients’ overall survival status and time and heatmap of the integrated model profiles. Rows represent clinical information and drug resistance probability, and columns represent patients. The grey dotted line represents the median integrated model risk score cutoff dividing patients into low- and high-score groups. **b** The Kaplan–Meier curves for patients in the validating dataset. The two-sided Log-rank test was performed to test the difference for OS between the high-risk and low-risk groups determined based on the median risk score from the validating set patients. The number of patients at risk was listed below the survival curves. The tick marks on the Kaplan–Meier curves represents the censored subjects. **c** The ROC curve had an AUC of 0.67

To test the robustness and repeatability of our findings, we validated our integrated gene- and clinical-signature in an independent dataset. Due to lack of validation, the model for PFS will not be discussed. We classified patients into high risk (n = 171) or low risk group (n = 171) using the median value of the risk scores generated with the same model formula above as cutoff. The distribution of the prognosis score (OS), the survival status of the DLBCL patients treated with CHOP-like chemotherapy and the gene expression

were shown in Fig. 2a. In accordance with the finding in the training dataset, patients with high risk had significantly shorter OS than individuals in low risk group (HR = 3.47; 95% CI 2.42–4.97; *p *= 1.53 × 10^−11^, by log-rank test, Fig. 2b, Additional file 11: Table S7). The ROC curves suggested that the prognosis score showed prediction power in predicting OS in the validating dataset (AUC = 0.67, Fig. 2c). The mean C-index 0.71 with a SE of 0.02 and significant RMS time ratio (1.97) showed integrated model for OS also had a stable predictive power in validating dataset. (Additional file 12: Table S8; Additional file 13: Table S9). And the integrated model represented higher HR value (HR = 3.31) than IPI (HR = 2.72), indicating that our integrated model performed a stronger power to predict patients’ survival (Additional file 14: Figure S5).

### Validation of the integrated gene-clinical signature in molecular subsets

**Fig. 3:**
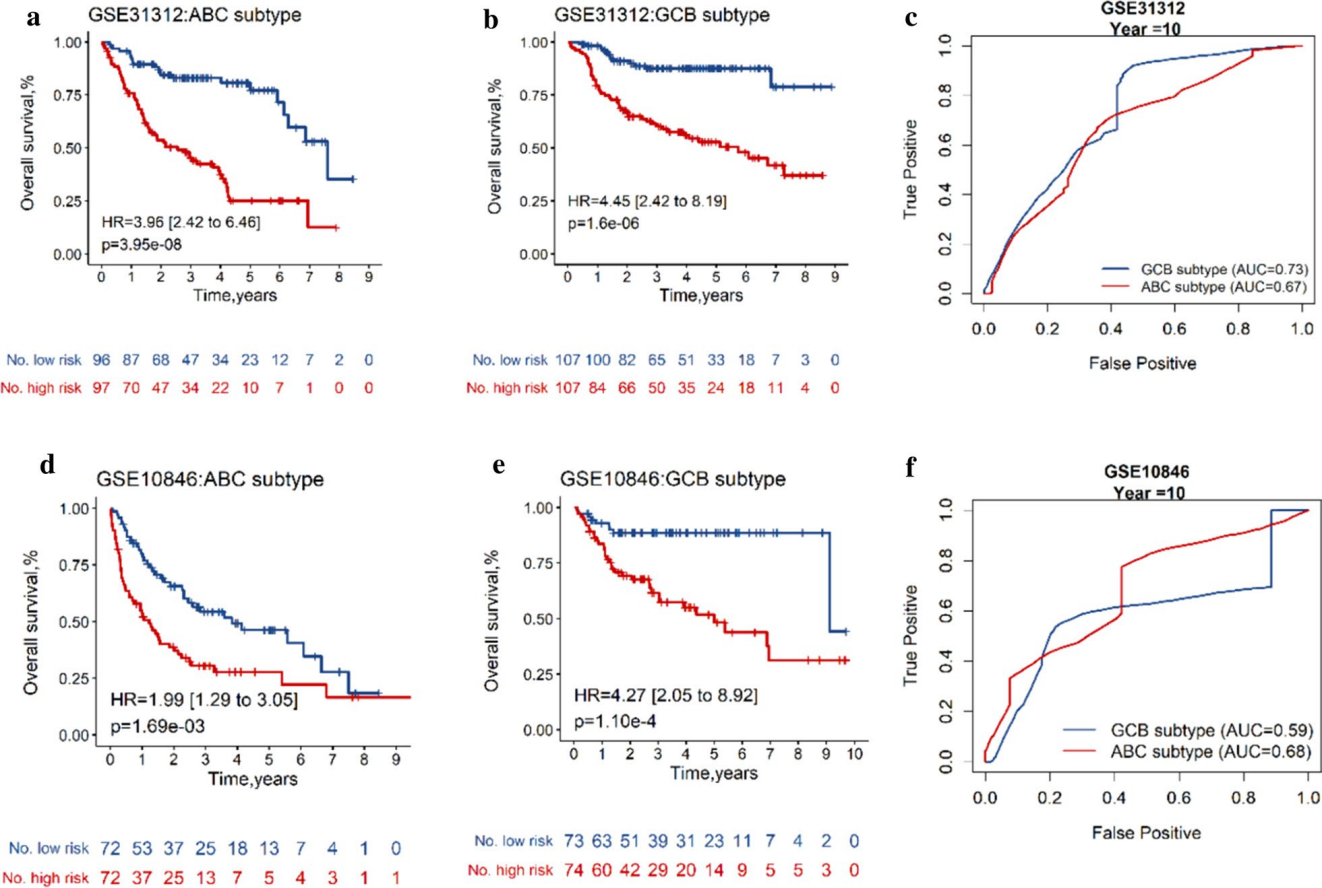
Integrated model performance for OS in ABC and GCB molecular subtypes. Kaplan–Meier curves with hazard ratio (HR), 95% confidence interval (CI) and log-rank p value for overall survival in the training cohort (**a**, **b**) and validating dataset (**d**, **e**) stratified by integrated model for OS into high and low risk. The ROC curves of the integrated model for prediction of OS in molecular subtypes in training dataset (**c**) and validating dataset (**f**)

A subgroup analysis was performed in different subsets according to the microarray molecular classifier for prognosis. In the training dataset, the prognosis score (OS) classified 199 patients with ABC subtype into high risk and low risk groups with obviously distinct OS (HR = 3.96, 95% CI 2.42–6.48, *p* = 3.95 × 10^−6^, by log-rank test, AUC = 0.67, Fig. 3a, c). As for 227 patients with GCB subtype, the forecast capability of the integrated signature between different risk subgroups in predicting OS (HR = 4.45, 95% CI 2.42–8.19, *p* = 1.6 × 10^−6^, by log-rank test, AUC = 0.73, Fig. 3b, c) was similar. In the validating dataset, patients with higher level of risk score were associated with shorter OS in ABC subtype (HR = 1.99, 95% CI 1.29–3.05, *p *= 1.69 × 10^−3^, by log-rank test, Fig. 3d, f) and GCB subtype (HR = 4.27, 95% CI 2.05–8.92, *p *= 1.10 × 10^−4^, by log-rank test, Fig. 3e, f). A subgroup analysis was also performed in different molecular subsets for PFS. In the training dataset, patients in different risk groups shown obviously distinct PFS either in ABC subtype (HR = 2.27, 95% CI 1.47–3.50, *p* = 1.98 × 10^−4^, by log-rank test, AUC = 0.62, Additional file 15: Figure S6) or GCB subtype (HR = 3.31, 95% CI 1.93–5.70, *p *= 1.52 × 10^−5^, by log-rank test, AUC = 0.70, Additional file 15: Figure S6). The results shown that the integrated gene-clinical model for OS or PFS has a prognostic value in both different subsets categorized based on a microarray molecular classifier.

## Discussion

DLBCL is a molecular heterogeneous disease leading to heterogeneous responses to therapy and different survival outcomes. Given the variations of drug resistance and clinical features among patients with DLBCL, the gene expression profiles (GEP) of tumor cells and clinical parameters have enabled the identification of genetic and clinical factors associated with prognostic value. In our study, we built a comprehensive DLBCL prognosis model for OS that combined 4 clinical factors and 2 genomic signatures of drug resistance. The prognostic probability of this integrated model was confirmed in another dataset. The results suggest that the combined genetic and clinical signature plays a crucial role in predicting prognosis of DLBCL and guiding therapeutic efficacy.

Numerous clinical, pathological and molecular markers have been developed to characterize the disease [2, 27–31]. Based on these characteristics, new different combinations of predictive biomarkers are being investigated to optimize the treatment and improve the outcomes of DLBCL. So far, there have been proposed advanced methods for survival prediction of various cancer and analysis of gene expression profiling. Some integrated bioinformatics analyses have progressively attempted to stratify patients with related diseases into low and high-risk groups, identified by gene expression profiling. Similar studies have also been reported for DLBCL. Caroline Bret et al. [32]. Shipp et al. [33] and Rosenwald et al. [34] have proposed several gene signatures to predict the clinical outcomes of DLBCL patients. Song et al. [35] demonstrated that CD59 could predict the reaction to R-CHOP treatment of patients with DLBCL. Another study identified a single molecular biomarker associated with diagnosis and treatment of DLBCL by using bioinformatics analysis [36]. Though molecular researches have filtered a plenty of genes, come out very divergent results. There are a few overlap prognostic genes in those studies. As we known, based on GEP is one of the promising methods for establish prognostic models to predict patients’ survival with high accuracy, this method is still having some limitations in clinical practice. While some GEP studies [24, 32–34, 37] showed that using genetic signatures could predict the clinical outcomes of DLBCL patients, lacking of reliable, stable and efficient biomarkers restrained its clinic application. Some studies demonstrating combined predictive models for prognosis is superior to predictive models relying on a single predictor [22, 38]. Combined gene biomarker of DLBCL and IPI score showed a better predictive capability for prognosis than either one of these markers alone [39]. Liu et al. [40] reported that combining gene signature (five-miRNA signature) and clinical factors (TNM stage) was a more sensitive predictor for nasopharyngeal carcinoma. In another study, the FGD3-SUSD3 metagene model was demonstrated to have a superior prognostic value for breast cancer [41].

To overcome the above problems, first we respectively selected genetic and clinical factors associated with OS of 449 patients by using univariate survival analysis in the training dataset. To improve the validity of the model, then we developed a promising prognostic signature model contained gene and clinical factors by using elastic net-regulated Cox regression. Interestingly, when combined gene signature with clinical signature, the integrated model led to a more potent prognostic prediction of DLBCL patients. And the integrated model seems to have a better predictive value for OS than IPI. Integrated model could probably ensure valid therapy for each individual patient before the treatment is initiated. In this study, we split DLBCL patients into two distinct risk groups according to the risk score of each individual, and we found that, as the time passing by, patients in low score group had better survival outcomes. In order to have a good prognosis for DLBCL patients, the patients with high risk score should tried other different treatment methods.

In recent years, activated B-cell-like (ABC) type and germinal center B-cell-like (GCB) type as two distinct molecular subtypes of DLBCL have been recognized by gene expression profiling, which based on Cell-of-Origin (COO) classifier [42]. These two molecular subclasses express different gene expression, clinical presentation and drug response [34, 43, 44]. Next, we performed the predictive power of our model in patients of ABC and GCB subtypes from training and validation datasets, the model’s prognostic capability was also strong in each group, indicating the good reproducibility and reliability of this signature. Though this signature showed few differences in the predictive capability, it is still powerful in both subtypes.

It is the first time to propose the idea of associating clinical information with drug resistance signatures to predicting prognosis of DLBCL individual with CHOP-like treatment. Nevertheless, there were several limitations in our study. First, we used only two independent datasets to respectively establish and validate the model. Therefore, more research is needed to validate this integrated model among enough number of DLBCL patients. Besides, there was only one drug resistance signature was independent of clinical factors, which may be caused by the limited sample number in analysis. And there were only three drug-specific resistance probability calculated in our study. The other drug resistance signatures may also worthy of investigation which need further research.

## Conclusions

In conclusion, the new comprehensive clinical-gene model for OS presented in our study has a better value than gene only model for predicting prognosis for patients with DLBCL. And these clinical information and drug-specific resistance probability of patients with DLBCL before treatment play a vital role in therapy management. Our findings suggest that combining clinical factors with genomic data could help us in-depth understand DLBCL survival and improve prognosis accuracy and promote the development of individualized therapy. Future investigations will focus on the validation of our defined integrated signature in planned clinical trials.

## Supplementary information

**Additional file 1: Table S1.** Summary of publically available DLBCL microarray datasets.

**Additional file 2: Table S2.** Univariate Cox regression analysis of overall survival and ridge regression coefficients of clinical information alone in the training dataset.

**Additional file 3: Table S3.** Univariate cox regression analysis of progression-free survival and ridge regression coefficients of clinical information or drug resistance signatures alone in the training dataset.

**Additional file 4: Table S4.** Univariate Cox regression analysis of overall survival and ridge regression coefficients of drug resistance signature alone in the training dataset.

**Additional file 5: Figure S1.** Pharmacogenomic model and Clinical model performance for overall survival in training and validating datasets. Kaplan–Meier curves with HR, 95% CI and log-rank p value for overall survival in the training dataset stratified by Pharmacogenomic model (**a**) and Clinical model (**b**) into high and low risk. Kaplan–Meier curves for overall survival in the validation dataset stratified by Pharmacogenomic model (**c**) and Clinical model (**d**) into high and low risk.

**Additional file 6: Figure S2.** The performance of Pharmacogenomic model and Clinical model for progression-free survival in training dataset. Kaplan–Meier curves with HR, 95% CI and log-rank p value for progression-free survival in the training dataset stratified by Clinical model (**a**) and Pharmacogenomic model (**b**) into high and low risk.

**Additional file 7: Table S5.** Multivariate Cox regression analysis of progression-free survival in training dataset.

**Additional file 8: Figure S3.** Cross-validation error curve. The left vertical dotted line reveals the partial likelihood deviance achieves its minimum at lambda = 0.0038, which represents a fairly regularized model (n = 6). The right vertical dotted line indicates the most regularized model (i.e., null model) with cross-validation error within one standard deviation of the minimum. The numbers at the top of the figure indicate the number of nonzero coefficients.

**Additional file 9: Table S6.** Univariate cox regression analysis of progression-free survival and ridge regression coefficients of clinical information and drug resistance signatures in the training dataset.﻿

**Additional file 10: Figure S4.** Integrated model analysis of patients for progression-free survival in the training dataset. Patients’ progression-free survival status and risk score generated with integrated model in the training dataset (GSE31312, n = 449). (**a**) The distribution plot, patients’ progression-free survival status and time and heatmap of the integrated model profiles. Rows represent clinical information and drug resistance probability, and columns represent patients. The grey dotted line represents the median integrated model risk score cutoff dividing patients into low- and high-score groups. (**b**) The Kaplan–Meier curves for patients in the training dataset. The two-sided Log-rank test was performed to test the difference for PFS between the high-risk and low-risk groups determined based on the median risk score from the training set patients. The number of patients at risk was listed below the survival curves. The tick marks on the Kaplan–Meier curves represents the censored subjects. (**c**) The ROC curve had an AUC of 0.68.

**Additional file 11: Table S7.** Kaplan–Meier analysis of overall survival in each dataset.

**Additional file 12: Table S8.** Restricted mean survival (RMS) analysis for model of overall survival between low- and high-risk groups in each dataset.

**Additional file 13: Table S9.** Incremental values of pharmacogenomic factors when added to the clinical factors for overall survival.

**Additional file 14: Figure S5** Kaplan–Meier curves of diffuse large B-cell lymphoma patients according to Integrated model for overall survival and International prognostic index (IPI) in training dataset (N = 415) and validating dataset (N = 302). Kaplan–Meier curves with HR, 95% CI and log-rank p value for overall survival in the training dataset stratified by Integrated model (**a**) and IPI (**b**) into high and low risk. Kaplan–Meier curves for overall survival in the validation dataset stratified by Integrated model (**c**) and IPI (**d**) into high and low risk.

**Additional file 15: Figure S6.** The Integrated model for progression-free survival performance in ABC and GCB molecular subtypes in training dataset. The ROC curves of the integrated model for prediction of PFS in molecular subtypes in training dataset (**a**). Kaplan–Meier curves with hazard ratio (HR), 95% confidence interval (CI) and log-rank p value for progression-free survival in the training dataset (**b**, **c**) stratified by integrated model into high and low risk.

## Data Availability

The datasets supporting the conclusions of this article are available in the in public databases Gene Expression Omnibus (https://www.ncbi.nlm.nih.gov/gds/) with the accession numbers: GSE31312 and GSE10846. All those studies previously were approved by their respective institutional review boards.

## Abbreviations

DLBLC: Diffuse large B-cell lymphoma
OS: Overall survival
PFS: Progression-free survival
HR: Hazard ratio
CI: Confidence interval
GEO: Gene expression omnibus
ROC: Receiver operating characteristic
AUC: Area under the receiver operating characteristic curve
ECOG: Eastern cooperative oncology group
GEP: Gene expression profiles
ABC: Activated B-cell-like
GCB: Germinal center B-cell-like
COO: Cell-of-origin
REGSs: Resistance gene signatures
IPI: International prognostic index
C-index: Concordance statistic
IDI: Integrated discrimination improvement
NRI: Net reclassification improvement
SE: Standard error
RMS: Restricted mean survival
