# Supplementary figures and images for: An integrated prognosis model of pharmacogenomic gene signature and clinical information for diffuse large B-cell lymphoma patients following CHOP-like chemotherapy
Source: J Transl Med. 2020 Mar 30;18:144. doi: 10.1186/s12967-020-02311-1 (PMC7106727; doi:10.1186/s12967-020-02311-1)

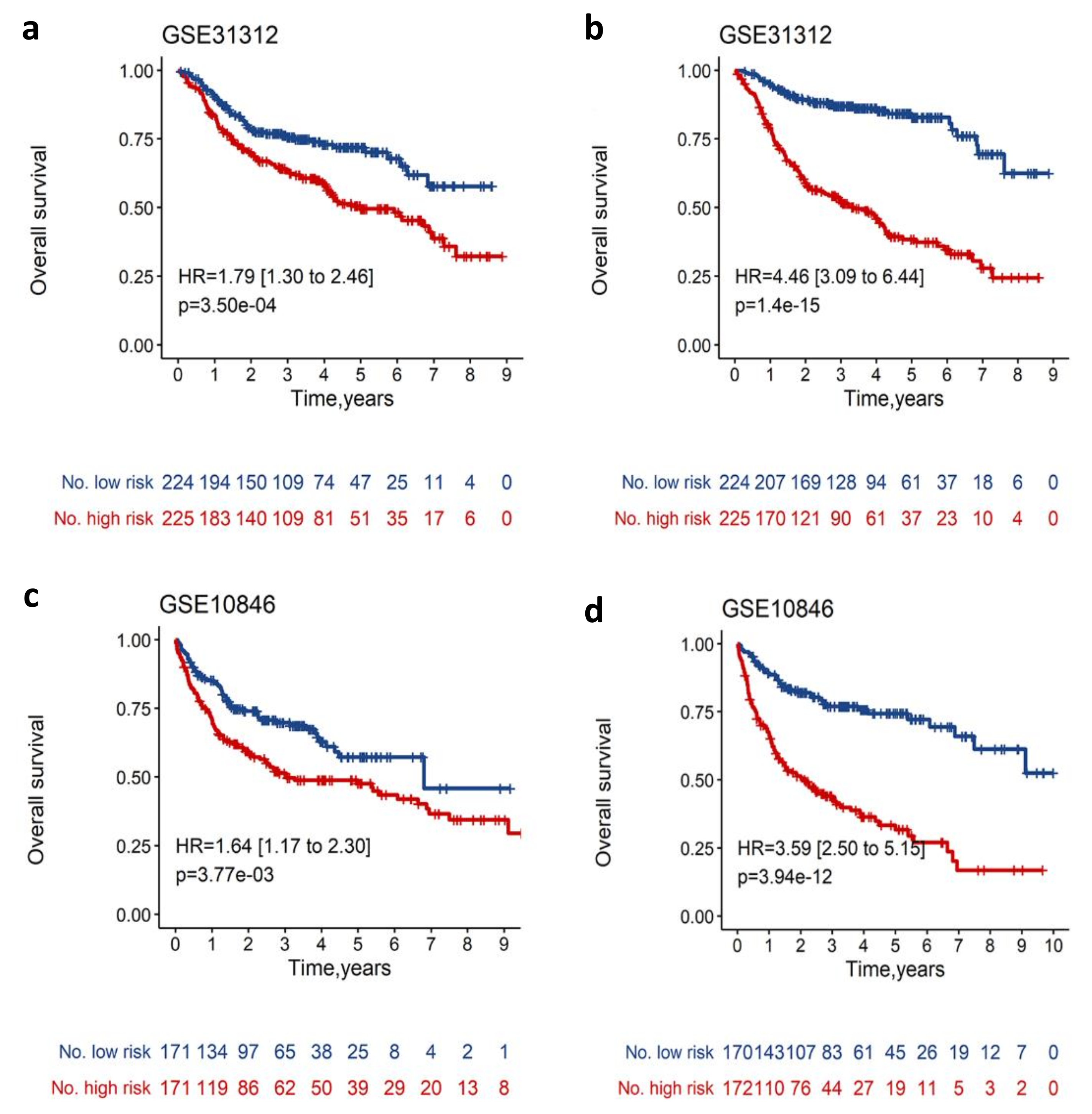

Supplement: Supplementary file 5 — Additional file 5: Figure S1. Pharmacogenomic model and Clinical model performance for overall survival in training and validating datasets. Kaplan–Meier curves with HR, 95% CI and log-rank p value for overall survival in the training dataset stratified by Pharmacogenomic model (a) and Clinical model (b) into high and low risk. Kaplan–Meier curves for overall survival in the validation dataset stratified by Pharmacogenomic model (c) and Clinical model (d) into high and low risk. [file 12967_2020_2311_MOESM5_ESM.tiff]

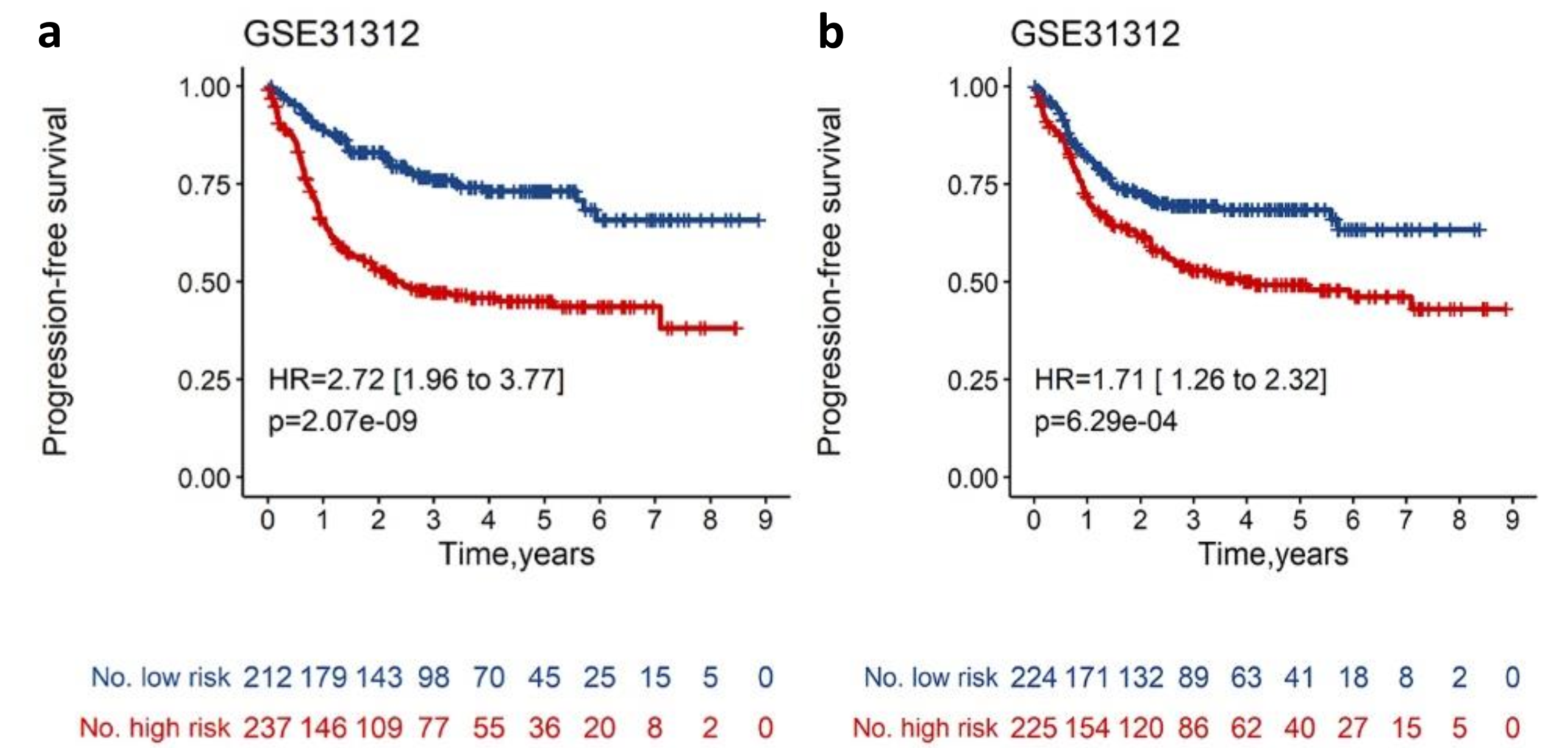

Supplement: Supplementary file 6 — Additional file 6: Figure S2. The performance of Pharmacogenomic model and Clinical model for progression-free survival in training dataset. Kaplan–Meier curves with HR, 95% CI and log-rank p value for progression-free survival in the training dataset stratified by Clinical model (a) and Pharmacogenomic model (b) into high and low risk. [file 12967_2020_2311_MOESM6_ESM.tiff]

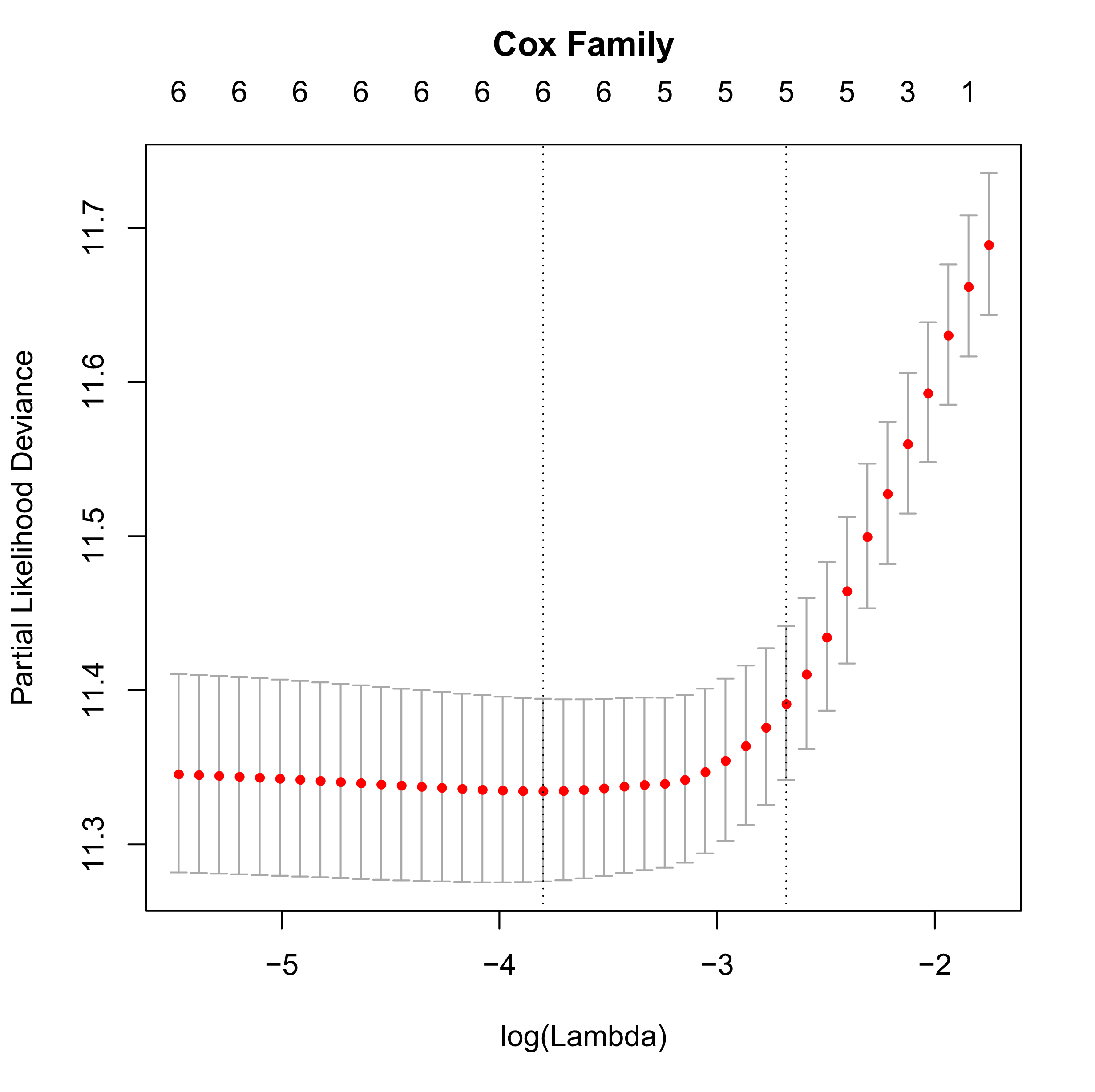

Supplement: Supplementary file 8 — Additional file 8: Figure S3. Cross-validation error curve. The left vertical dotted line reveals the partial likelihood deviance achieves its minimum at lambda = 0.0038, which represents a fairly regularized model (n = 6). The right vertical dotted line indicates the most regularized model (i.e., null model) with cross-validation error within one standard deviation of the minimum. The numbers at the top of the figure indicate the number of nonzero coefficients. [file 12967_2020_2311_MOESM8_ESM.tiff]

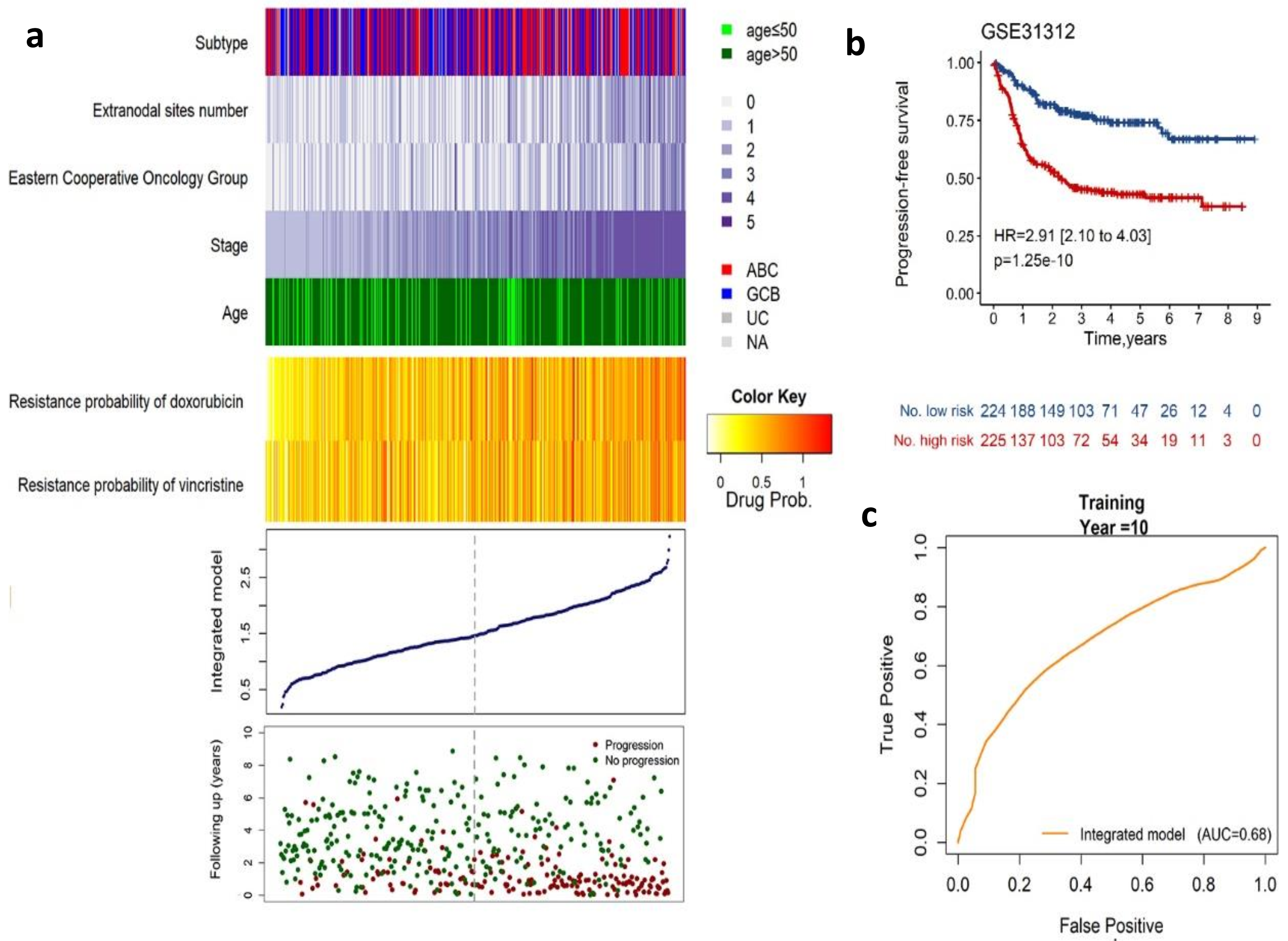

Supplement: Supplementary file 10 — Additional file 10: Figure S4. Integrated model analysis of patients for progression-free survival in the training dataset. Patients’ progression-free survival status and risk score generated with integrated model in the training dataset (GSE31312, n = 449). (a) The distribution plot, patients’ progression-free survival status and time and heatmap of the integrated model profiles. Rows represent clinical information and drug resistance probability, and columns represent patients. The grey dotted line represents the median integrated model risk score cutoff dividing patients into low- and high-score groups. (b) The Kaplan–Meier curves for patients in the training dataset. The two-sided Log-rank test was performed to test the difference for PFS between the high-risk and low-risk groups determined based on the median risk score from the training set patients. The number of patients at risk was listed below the survival curves. The tick marks on the Kaplan–Meier curves represents the censored subjects. (c) The ROC curve had an AUC of 0.68. [file 12967_2020_2311_MOESM10_ESM.tiff]

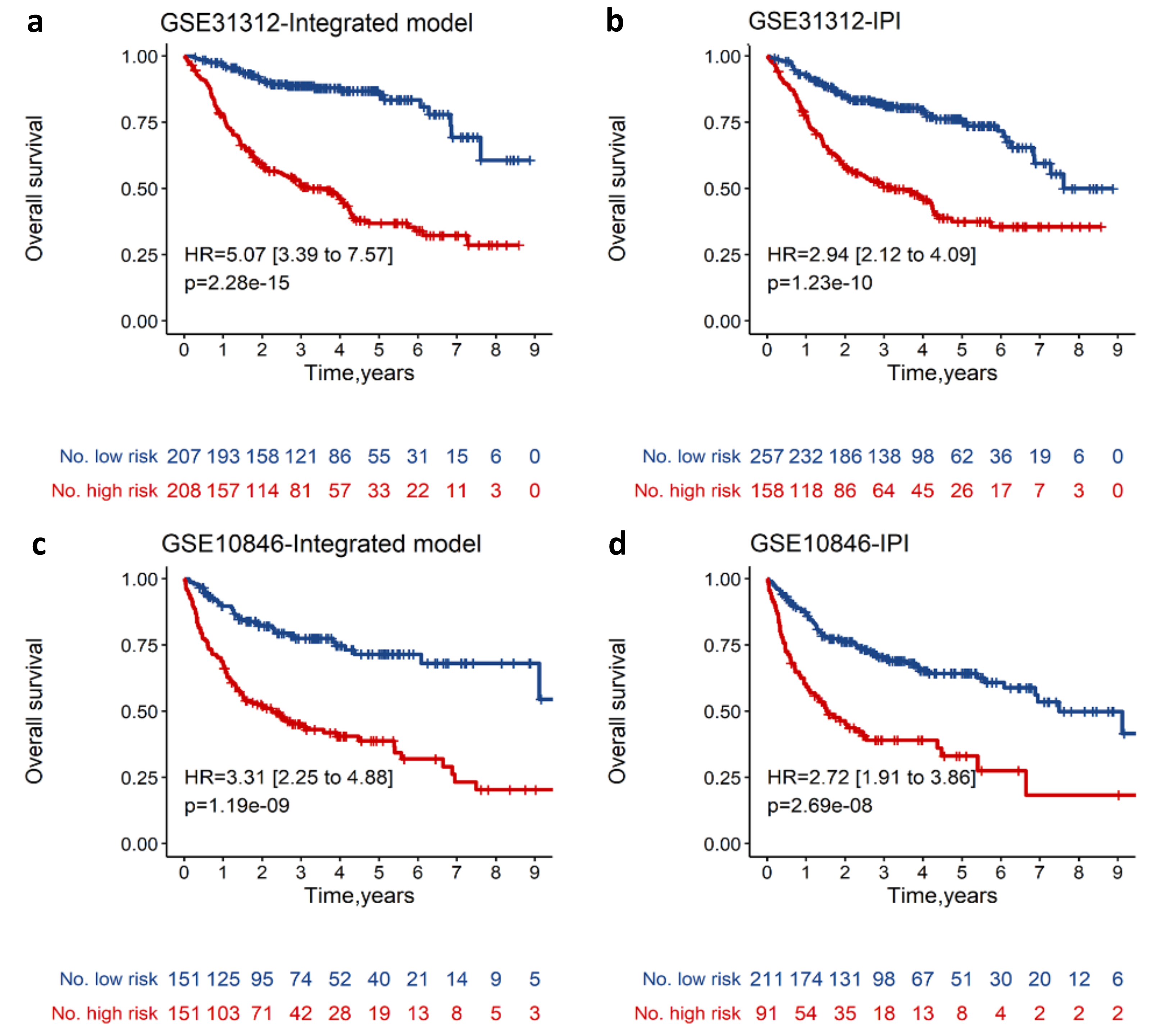

Supplement: Supplementary file 14 — Additional file 14: Figure S5 Kaplan–Meier curves of diffuse large B-cell lymphoma patients according to Integrated model for overall survival and International prognostic index (IPI) in training dataset (N = 415) and validating dataset (N = 302). Kaplan–Meier curves with HR, 95% CI and log-rank p value for overall survival in the training dataset stratified by Integrated model (a) and IPI (b) into high and low risk. Kaplan–Meier curves for overall survival in the validation dataset stratified by Integrated model (c) and IPI (d) into high and low risk. [file 12967_2020_2311_MOESM14_ESM.tiff]

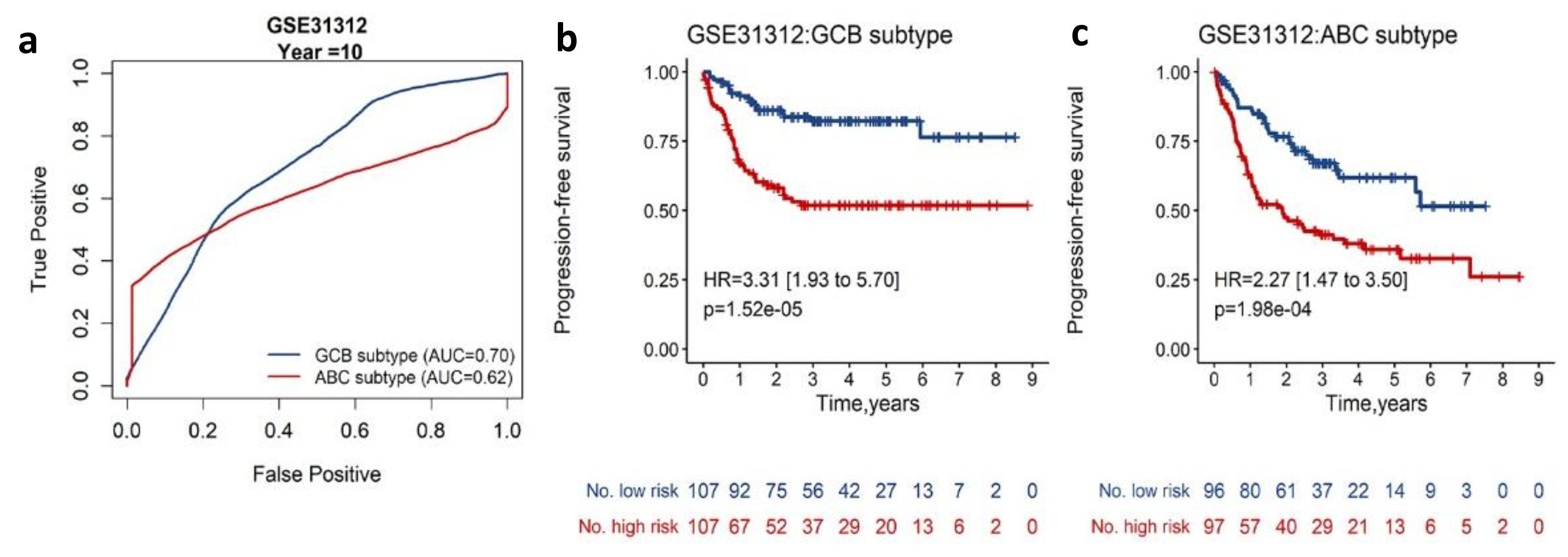

Supplement: Supplementary file 15 — Additional file 15: Figure S6. The Integrated model for progression-free survival performance in ABC and GCB molecular subtypes in training dataset. The ROC curves of the integrated model for prediction of PFS in molecular subtypes in training dataset (a). Kaplan–Meier curves with hazard ratio (HR), 95% confidence interval (CI) and log-rank p value for progression-free survival in the training dataset (b, c) stratified by integrated model into high and low risk. [file 12967_2020_2311_MOESM15_ESM.tiff]
